# Stress susceptibility-specific phenotype associated with different hippocampal transcriptomic responses to chronic tricyclic antidepressant treatment in mice

**DOI:** 10.1186/1471-2202-14-144

**Published:** 2013-11-13

**Authors:** Pawel Lisowski, Grzegorz R Juszczak, Joanna Goscik, Adrian M Stankiewicz, Marek Wieczorek, Lech Zwierzchowski, Artur H Swiergiel

**Affiliations:** 1 Department of Molecular Biology, Institute of Genetics and Animal Breeding, Polish Academy of Sciences, Postepu 36A, 05-552, Magdalenka, Jastrzebiec n/Warsaw, Poland; 2 Department of Animal Behavior, Institute of Genetics and Animal Breeding, Polish Academy of Sciences, Postepu 36A, Jastrzebiec n/Warsaw, Poland; 3 Department of Software Engineering, Bialystok Technical University, Wiejska 45A, Bialystok, Poland; 4 Department of Neurobiology, Faculty of Biology and Environmental Protection, University of Lodz, Pomorska 141/143, Lodz, Poland; 5 Department of Animal and Human Physiology, Institute of Biology, Gdansk University, Wita Stwosza 59, Gdansk, Poland; 6 Department of Pharmacology, Toxicology and Neuroscience, Louisiana State University Health Sciences Center, 1501 Kings Highway, Shreveport, USA

## Abstract

**Background:**

The effects of chronic treatment with tricyclic antidepressant (desipramine, DMI) on the hippocampal transcriptome in mice displaying high and low swim stress-induced analgesia (HA and LA lines) were studied. These mice displayed different depression-like behavioral responses to DMI: stress-sensitive HA animals responded to DMI, while LA animals did not.

**Results:**

To investigate the effects of DMI treatment on gene expression profiling, whole-genome Illumina Expression BeadChip arrays and qPCR were used. Total RNA isolated from hippocampi was used. Expression profiling was then performed and data were analyzed bioinformatically to assess the influence of stress susceptibility-specific phenotypes on hippocampal transcriptomic responses to chronic DMI. DMI treatment affected the expression of 71 genes in HA mice and 41 genes in LA mice. We observed the upregulation of *Igf2* and the genes involved in neurogenesis (HA: *Sema3f*, *Ntng1*, *Gbx2*, *Efna5*, and *Rora*; LA: *Otx2*, *Rarb*, and *Drd1a*) in both mouse lines. In HA mice, we observed the upregulation of genes involved in neurotransmitter transport, the termination of GABA and glycine activity (*Slc6a11*, *Slc6a9*), glutamate uptake (*Slc17a6*), and the downregulation of neuropeptide Y (*Npy*) and corticotropin releasing hormone-binding protein (*Crhbp*). In LA mice, we also observed the upregulation of other genes involved in neuroprotection (*Ttr*, *Igfbp2, Prlr*) and the downregulation of genes involved in calcium signaling and ion binding (*Adcy1*, *Cckbr, Myl4, Slu7, Scrp1, Zfp330*).

**Conclusions:**

Several antidepressant treatment responses are similar in individuals with different sensitivities to stress, including the upregulation of *Igf2* and the genes involved in neurogenesis. However, the findings also reveal that many responses to antidepressant treatments, involving the action of individual genes engaged in neurogenesis, neurotransmitter transport and neuroprotection, depend on constitutive hippocampal transcriptomic profiles and might be genotype dependent. The results suggest that, when and if this becomes feasible, antidepressant treatment should take into consideration individual sensitivity to stress.

## Background

The hippocampus is an integral component of the limbic system and mechanisms that control mood [1,2]. The hippocampus also plays an important role in stress, pain and nociceptive responses and might mediate depressive behaviors [3]. Because the propensity to develop depression and the responses to antidepressant treatments might depend on the susceptibility of an individual to stress, we studied mice selected for high (HA line) or low (LA line) stress reactivity, as measured by the magnitude of swim stress-induced analgesia (SIA) [4]. There were differences among the mice in terms of responses to a variety of antidepressants (desipramine, venlafaxine, and aminosenktide) and profound differences in tail suspension and forced swim tests, reflecting depression-like behaviors [5-7]. Under control conditions, clear differences in the constitutive, hippocampal transcriptomic profiles related to the upregulation of genes involved in calcium signaling in LA mice and the robust upregulation of genes encoding receptors for neurotensin (*Ntsr2*) and GABA (*Gabard*) in HA mice were observed [8]. These findings suggested that the selective breeding for swim SIA affected many aspects of hippocampal neuron physiology, including metabolism, structural changes and cellular signaling. Furthermore, unpredictable chronic mild stress (CMS), used to trigger desipramine-reversible depressive-like behavior [9], produced mouse line-specific responses in hippocampal gene expression [10]. The results suggest that hippocampal responses to stress depend on genotype, and the potential drug targets against the detrimental effects of stress might include glutamate transport, neurogenesis, axonogenesis, neurite development, or chromatin modifications, to name a few.

The present study was conducted to identify transcriptomic response involved in the action of the tricyclic antidepressant, desipramine, in the hippocampus. We used expression microarray analyses to characterize changes in the transcription profiles and identify genes that are affected after chronic treatment with DMI in the hippocampus of the two distinct mouse lines selected for stress reactivity. The results provide insight into the genetic background that might modulate the molecular response to antidepressants and mediate resistance to antidepressants.

## Methods

### Animals

Swiss-Webster male mice (weighing 25–30 g, 12 weeks old) exhibiting high (HA) and low (LA) swim stress-induced analgesia (SIA) were used [4]. Briefly, the outbred male and female mice were scored for the latency of the hind paw nociceptive reflex on a 56°C hot plate at 2 min after the completion of a 3-min swimming test in 20°C water. The animals displaying the longest (50 – 60 s) and the shortest (<10 s) post-swim latencies of the nociceptive response were selected as progenitors of the HA and LA lines. A similar procedure was repeated in subsequent offspring, and the animals displaying the longest and the shortest post-swim hot plate latencies were mated to maintain the lines or selected for use in subsequent experiments. The HA and LA male mice used in the present experiment belonged to the 68th generation of albino Swiss–Webster mice that have been selectively bred in our laboratory for high (HA line) and low (LA line) magnitudes of swim-stress induced analgesia (SSIA) as described earlier [4].

Animals were males, 5 months old and weighed 41.5 ± 0.8 g and 39.5 ± 0.5 g (HA and LA lines, respectively). Mice from each line and treatment group were housed four to six per cage on a 12-h/12-h light dark cycle with unlimited access to standard murine chow food and water. All procedures were carried out between 9 am and 4 pm. Mice from HA and LA lines were injected and decapitated in a pseudorandom order.

### Treatment

HA and LA animals were injected intraperitoneally with desipramine (DMI, 7.5 mg/kg/day, Sigma-Aldrich, USA) or saline once a day for 21 consecutive days. of the experiment. Each experimental group contained 12 mice. DMI was selected based on the results obtained from our previous studies [5,6]. These studies revealed that DMI shortened the immobility period of HA mice in the forced swim and tail suspension tests but was ineffective in LA animals. All procedures involving animals were performed in accordance with the Guiding Principles for the Care and Use of Research Animals, and were approved by the the Institutional Ethics Committee of the Institute of Genetics and Animal Breeding and then by the National Third Local Ethics Commission (permission No. 3/2005). Permission had been issued in accordance with the guidelines of the pertinent Polish Parliamentary Bill. All observations were performed by a trained observer who was blinded to the treatment conditions and was not informed of the treatment conditions in advance.

### Samples preparation

Two days after the injections, the animals were sacrificed through decapitation (HA DMI, n = 12; HA saline, n = 12; LA DMI, n = 12; LA saline, n = 12), and the brains were rapidly removed. The hippocampi were quickly dissected on ice-cold glass, inserted into freezing vials, frozen in liquid nitrogen and stored at -80°C until further analysis. Total RNA was isolated separately from each individual hippocampus using the NucleoSpin RNA II kit (Macherey-Nagel, Germany). A Nanodrop (Nanodrop, USA) and Bioanalyzer (Agilent, USA) were used to estimate the quantity and quality of each RNA sample, and the RIN (RNA Integrity Number) values ranged from 9.2 to 9.8 for all samples.

To minimize the influence of the individual differences between the animals and the variation introduced through dissection and tissue preparation, the individual RNA samples from three mice were pooled. The quantity and quality of the RNA in the pools were estimated using a Nanodrop and Bioanalyzer. Each pool was subsequently converted to biotinylated cRNA using the Illumina RNA Amplification Kit (Ambion Inc., USA) and 100 ng of total RNA, purified using the RNeasy kit (Qiagen, Germany) and hybridized to a single microarray. Four independent biological replicates of the microarray analysis were prepared for each group of mice.

### Microarray, hybridization and fluorescent detection

The gene expression profiling was performed using MouseRef-8_V2 Expression BeadChip microarrays (Illumina, USA) according to MIAME guidelines [11]. Each array contains >22,000 well-annotated RefSeq 50-mer oligonucleotide probes selected from the NCBI database (Release 16). The cRNA samples were applied to the arrays and assembled onto hybridization cartridges. Overall, 16 hybridizations (4 for each experimental group) were performed. The hybridization and washing of the arrays was performed according to the manufacturer’s hybridization protocol. The microarrays were scanned on a BeadArray Reader (Illumina, USA).

### Data normalization, selection of the most differently expressed genes and hierarchical clustering

Raw microarray data were processed using the BeadArray and LIMMA package of the R statistical language (Bioconductor project; [12]). The data preprocessing involved the normalization of the expression levels using a quantile method preceded by log2 transformation. Linear model fitting was performed for the pre-processed dataset. The coefficients of the obtained fitted models described the differences between the RNA sources hybridized to the arrays. The empirical Bayesian analysis was performed to determine whether the contrast coefficients from the linear models were equal zero and identify differentially expressed genes. The genes showing log fold-changes greater than 0.5 (logFC > 0.5) and p < 0.05 were considered significantly differentially expressed. The Benjamini and Hochberg method [13], to control the false discovery rate (FDR), was used to correct the p values.

### Gene lists enrichment analysis

The gene lists (GenBank accession numbers) generated from the microarray results were submitted to the Database for Annotation, Visualization and Integrated Discovery (DAVID) v6.7. [14]. The genes were classified into functional categories using biological process (BP), cellular components (CC), and molecular function (MF) ontologies. Analysis of the association of the genes with physiological or biochemical pathways was performed using the Kyoto Encyclopedia of Genes and Genomes database (KEGG; [15]). To identify significantly overrepresented biological categories and KEGG pathways within the lists of differentially expressed genes, the threshold for the enrichment analysis was set at p ≤ 0.05. For details see Lisowski et al., 2011 [10,16].

### Quantitative real-time PCR (qPCR) for microarray validation

To validate the results of the microarrays, quantitative real-time PCR (qPCR) with SYBR Green was performed as previously described [10], according to MIQE guidelines [17]. Eight genes, belonging to different functional categories and significantly differing in the expression between the saline and desipramine experimental groups, were selected in each line. The qPCR assays were performed in triplicate on the same, non-pooled individual RNA samples, which were used in the microarray experiment. All amplified PCR fragments were sequenced to verify the resulting PCR product.

For reference, using a previously described methodology [18,19], 10 common reference genes belonging to different functional classes *(Gapdh, Tbp, Hprt1, Actb, B2m, Rpl13a, Hsp90ab1, Gusb, Tfrc,* and *Ppia*) were assessed using qPCR, and two housekeeping genes stably expressed in hippocampi of DMI-treated mice were used: TATA box binding protein (*Tbp*) and hypoxanthine guanine phosphoribosyl transferase 1 (*Hprt1*). The normalization factor (NF) was obtained from the geometric mean of the raw expression data of *Tbp* and *Hprt1*.

The primers were designed using ExonPrimer software (Institute of Human Genetics, TUM/Helmholtz Center Munich, Germany; [20]) and *Mus musculus* GenBank sequences. All primers produced amplicons spanning two exons in highly conserved coding regions and included all known alternatively spliced mRNA variants. Additional file 1 summarizes the primer information, including gene names, primer sequences, amplicon lengths, annealing temperatures, and GenBank accession numbers.

The data from the triplicate qPCR reactions were normalized using the average cycle threshold (Ct) value and a mathematical model for the relative quantification of the real-time RT-PCR results [21]. The qPCR data obtained from the saline and DMI-treated groups were analyzed using t-tests. The differences between the groups were considered significant at p < 0.05. The degree and significance of the correlation between fold-changes, as determined in the microarray and qPCR analyses, were evaluated using the Pearson moment correlation.

## Results

### Genome-wide gene expression

Comparisons of the hippocampal transcriptomic profiles of the control and desipramine-treated animals revealed that DMI affected the expression of 71 genes in HA and 41 genes in LA mice, meeting the criteria of log fold-changes greater than 0.5 and adjusted p values <0.05. DMI treatment in HA mice, compared with saline injections, resulted in the upregulation of 57 transcripts and the downregulation of 14 transcripts. DMI treatment in LA animals resulted in upregulation of 28 transcripts and downregulation of 13 transcripts. The entire set of probes, with the corresponding gene names and the FC and p values that differed between the saline- and DMI-treated animals are presented in Additional files 2 and 3. All genes annotated to these probe sets were expressed in mouse hippocampus according to the Novartis Gene Expression Atlas [22]. The cell-type classification analysis of differentially expressed genes, according to Cahoy [23], the GeneCards® database [24] screening and Ingenuity Pathway Analysis® [25] of the major canonical pathways, revealed that most of these genes are characteristic of neurons and oligodendrocytes.

### Effect of desipramine on hippocampal transcriptome in HA line

DMI treatment in HA mice resulted in the upregulation of transcripts involved in 12 significant (p < 0.05) biological processes, 5 cellular components, 7 molecular function terms and 2 biochemical pathways. The biological process terms included neurotransmitter transport (p = 0.001) (*Slc6a9, Slc17a6, Slc6a11, Rims3*), cell motion (p = 0.002) (*Vav3, Sema3f, Gbx2, Efna5, Elmo1*), cell projection organization (p = 0.003) (*Vav3, Ntng1, Gbx2, Efna5*), angiogenesis (p = 0.005) (*Gbx2, Arhgap24, Anxa2*) axonogenesis/neuron differentiation/neuron projection (p = 0.004) (*Ntng1, Gbx2, Efna5, Rora*), behavior (p = 0.039) (*Ptgds, Sema3f, Chrna4, Zic1*) and neurogenesis (p = 0.048) (*Sema3f, Ntng1, Gbx2, Efna5, Rora*). The cellular component terms included elements of the cell junction (p = 0.0002) (*Cbln1, Slc17a6, Syt9, Chrna4, Arhgap24, Calb2, Anxa2, Rims3*), plasma membrane (p = 0.001) (*Slc6a11, Ntng1, Syt9, Arhgap24, Calb2, Rims3, Anxa2, Ly6a, Gng8, Slc6a9, Cbln1, Slc17a6, Chrna4*), and the synapse (p = 0.011) (*Cbln1, Slc17a6, Syt9, Chrna4, Rims3*). The molecular function terms included amino acid transmembrane transport (p = 0.007) (*Slc6a9, Slc17a6, Slc6a11*) and chemorepellent activity (p = 0.01) (*Sema3f, Efna5*). To identify significantly over-represented pathways in the list of DMI-upregulated genes, we searched the KEGG database. In the hippocampus of HA mice, the significantly overrepresented pathways included chemokine signaling (p = 0.003) (*Gng8, Vav3, Prkcd, Elmo1*) and axon guidance pathways (p = 0.021) (*Sema3f, Ntng1, Efna5*).

The downregulated transcripts were involved in 6 significant (p < 0.05) biological processes and 1 cellular component term, including mast cell (p = 0.009) and myeloid leukocyte activation (p = 0,02), the regulation of response to external stimulus (p = 0.023) and vesicle-mediated transport (p = 0.029) (*Cplx2, Fcer1g, Npy, Arc*) and coding elements of synapses (p = 0.026) (*Phactr1, Arc, Cplx2*).

Moreover, as shown in Additional file 1: Table S1, DMI treatment resulted in the upregulation of neuronatin (*Nnat*), a factor involved in the modulation of dendritic calcium levels during homeostatic plasticity, and the downregulation of neuropeptide Y (*Npy*), which is implicated in a diverse range of biological actions, including anxiolysis and the control of gonadotrophin-releasing hormone or pituitary hormone secretion. DMI also caused the downregulation of corticotropin-releasing hormone-binding protein (*Crhbp*), which inactivates CRH. For the list of GO functional category classifications of up- and down-regulated genes affected after DMI treatment in the HA line see Additional file 4.

### Effect of desipramine on hippocampal transcriptome in the LA line

The genes upregulated in LA mice after DMI treatment are involved in 13 significant (p < 0.05) biological processes and 2 cellular component terms. The biological process terms included behavior (p = 0.021), including behavioral interactions (p = 0.045), mating (p = 0.016), reproduction (p = 0.028) and locomotory behavior (p = 0.048) (*Pde1b, Ppp1r1b, Enpp2, Drd1a)*, brain structure development, including striatum (p = 0.015), subpallium (0.019), eye (p = 0.022) and forebrain (p = 0.025), and neurogenesis (p = 0.03) (*Otx2, Rarb, Drd1a*). The cellular component terms included elements of the extracellular space (p = 0.006) and extracellular region (p = 0.035) (*Ttr, Kl, Enpp2, Sostdc1, 1500015o10rik, Tac1, Igf2, Igfbp2*). The molecular function terms and biochemical pathways were not included in the list of upregulated genes in LA mice and were not statistically significant. The downregulated genes in the LA mice transcriptomes were not significantly enriched.

Notably, the transcripts upregulated after DMI treatment in LA mice include transthyretin (*Ttr*), the prolactin receptor (*Prlr*), insulin-like growth factor 2 (*Igf2*), and insulin-like growth factor binding protein 2 (*Igfbp2*), which are important genes involved in neuroprotection. For the list of GO functional category classifications of up- and down-regulated genes affected after DMI treatment in the LA line see Additional file 5*.*

### Quantitative real-time PCR (qPCR) for microarray validation

**Figure 1:**
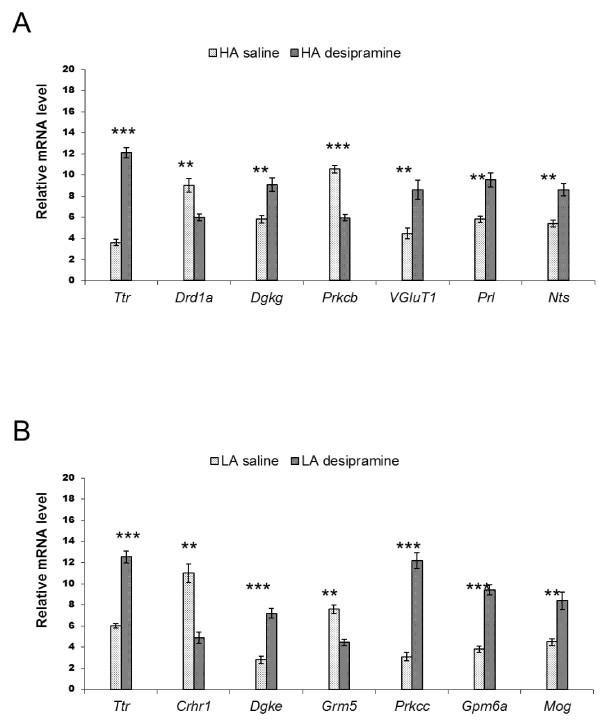
**Validation of expression of the selected genes by qPCR.** Saline-treated HA mice vs. desipramine-treated HA mice **(A)**; saline-treated LA mice vs. desipramine-treated LA mice **(B)**. Results are presented as means of relative mRNA levels in 12 individuals per experimental group; error indicators show ± S.E.M. Values differ significantly at *p < 0.05, **p < 0.01 or ***p < 0.001; qPCR values were normalized to geometric mean of the raw expression data of two reference genes: *Gapdh* and *Hprt1*. Abbreviations: qPCR, quantitative real-time RT-PCR; HA, high analgesia mice; LA, low analgesia mice; S.E.M., standard error of measurement or mean; *Tbp*, TATA box binding protein; *Hprt1*, hypoxanthine phosphoribosyltransferase 1.

To validate the microarray results, 16 (8 from HA and 8 from LA) differentially expressed genes (DEGs) were selected for quantification using qPCR. The DEGs were selected from among the significant functional groups associated with neurotransmitter transport, immunity, cell differentiation, neurogenesis, axonogenesis, and calcium signaling and axon guidance pathways. The following genes were selected for the HA line: *Chrna4, Crhbp, Gbx2, Igf2, Nnat, Npy, Slc6a11*, and *Slc17a6*. The following genes were selected for the LA line: *Cckbr, Csrp1, Drd1a, Igf2, Igfbp2, Otx2, Prlr*, and *Ttr*. Differences in the expression revealed using microarray analyses were confirmed. The expression levels are shown in Figure 1. The DEGs identified through microarray tests were consistent with the qPCR results (R = 0.94, p < 0.0001).

## Discussion

Early comparisons of the constitutive gene expression profiles between the HA and LA lines identified 205 transcripts with different levels of mRNA in the hippocampi of non-stressed animals [8]. The results suggest that individual differences in depression-like behavioral patterns may be associated with the characteristics of the constitutive hippocampal transcriptome.

The present study indicates that two different basal hippocampal transcriptomic profiles influenced the response to DMI, although common changes induced through drug treatments were also observed. The putative genes associated with the action of the ADs in the brain based on the literature search indicated that chronic AD treatments increase the levels of the cAMP response element-binding protein (CREB) mRNA and its receptor trkB in the hippocampus [26-28]. Furthermore, other transcription factors, such as Fos family members, are also increased in the brain after the chronic administration of ADs [29,30]. Antidepressant drugs produce antidepressant effects in the prefrontal cortex and hippocampus [31]. Experiments have demonstrated that antidepressants significantly affect the cAMP cascade, and a homologous pathway exists for all classes of antidepressants. However, other observations, using different biochemical and molecular assays, suggest limited changes. Thus, genes encoding cytochrome b [32], glyceraldehyde-3-phosphate dehydrogenase (GAPDH) [33], the RING-H2 finger motif gene [34], the cysteine string protein [35], the tetraspan protein [36], the GRB2 homeobox protein and the ribosomal protein L35a [37], isoforms of protein kinase C and cAMP-dependent protein kinase [38], the *Mss4* gene [39], VAMP2/synaptobrevin-2 [40], and GAP-43 [41] are regulated through treatment with antidepressants in different parts of the rodent brain. Long-lasting neuroadaptations likely include complex changes in the gene expression in the limbic system of the brain. For example, changes in the expression of *Crhbp* and *Npy* (downregulated in HA) after AD treatment have been reported [42]. Evidence of the importance of ion transport in the rat brain cortex has been shown in the context of antidepressant treatment [43,44]. Specifically, changes in gene expression coding elements of calcium and sodium channels were observed after 28 days of treatment with amitryptiline [45]. In the present study, downregulation of adenylate cyclase 1 (*Adcy1*) and the cholecystokinin B receptor (*Cckbr*) involved in calcium signaling occurred in the LA line. In the HA line, we observed the upregulation of neuronatin (*Nnat*). A recent study showed that Nnat adjusts dendritic calcium levels through the regulation of intracellular calcium storage, thus neuronatin might impact synaptic plasticity through the modulation of dendritic calcium levels during homeostatic plasticity, thereby potentially regulating neuronal excitability, receptor trafficking, and calcium dependent signaling in hippocampal neurons [46]. The results obtained in the present study, however, also implicate a number of novel “AD genes”. Among these, alterations in the expression of genes involved in neurogenesis (*Sema3f, Ntng1, Gbx2, Efna5, Rora, Otx2, Rarb, Drd1a*) and neuroprotection (*Ttr, Prl, Igf2*) are of particular interest.

### Effects of desipramine on genes related to neurogenesis

The chronic administration of antidepressants increases the proliferation and survival of neural stem cells and new neurons in the hippocampus [47-49]. Increased stem cell proliferation is required for the behavioral effects of antidepressants, suggesting that this effect might underlie the therapeutic action of antidepressant drugs on mood [50]. It takes weeks for newborn neurons to differentiate and mature into fully functional hippocampal granule neurons [51], suggesting that the gradual maturation of newly born dentate granule neurons might explain the delayed action of antidepressant drugs [52]. Specific gene expression profiles of neurogenesis in AD treatment have not been clearly identified, although according to Gene Ontology [53] classification, the upregulation of five genes in the HA line (*Sema3F, Ntng1, Gbx2, Efna5, Rora*) and three genes in the LA line (*Otx2, Rarb, Drd1a*) involved in neurogenesis, neurite outgrowth, and the proliferation and differentiation of neuronal progenitor cells were detected. *Igf2* should be added to the group of genes related to neurogenesis, as this gene was upregulated in both lines after DMI treatment.

### Effects of desipramine on genes related to the chronic stress response

The analyses performed in the present work indicated a robust upregulation of *Ttr* gene-coding transthyretin (TTR) and the *Prlr* gene-coding prolactin receptor in the LA line, and in both lines, we observed the upregulation of the *Igf2* gene encoding somatomedin. TTR is a carrier protein and a major transporter of thyroid hormones and retinol in plasma and cerebrospinal fluid [54]. The clinical features of defects in TTR include seizures, stroke-like episodes, dementia and psychomotor deterioration. However, the antidepressant-like effect of the histone deacetylase inhibitor sodium butyrate (SB) upregulates transthyretin (*Ttr*), with simultaneous increases in histone H4 acetylation at the promoter of the *Ttr* gene [55]. Moreover, the genes, including *Ttr, Prl* and *Igf2*, whose hippocampal expression patterns were downregulated in four rat models of endogenous depression and chronic stress, represent a generalized molecular response to chronic stress [56]. In addition, stress has previously been shown to increase the expression of neuropeptide Y [57-59], which in the present study was downregulated in the HA line after DMI treatment. Prolactin is a neuromodulator of behavioral and neuroendocrine stress in the rat [60,61]. The downregulation of brain prolactin receptors increased anxiety-related behavior, demonstrating an anxiolytic effect of PRL in the brain [61]. Furthermore the stress-induced increase of corticotropin secretion was reduced after the chronic intracerebroventricular infusion of PRL [61]. Fujikawa [62] showed that PRL levels increase in response to stress acting on the central nervous system to protect against acute stress-induced hypocalcemia. Moreover, a recent study on gene expression profiling in neural stem cells (NSCs) showed the upregulation of insulin-like growth factor 2 (*Igf2*) in the dentate gyrus (DG) compared with that in immature neurons. IGF2 selectively controls the proliferation of DG NSCs *in vitro* and *in vivo* through AKT-dependent signaling. Thus, the gene expression profiling of NSCs and progeny cells revealed that IGF2 is a novel regulator of adult neurogenesis [63]. Altogether, these findings suggest the possibility that the upregulation of transcripts for *Ttr, Prlr* in HA line, and *Igf2* in both lines might be involved in AD actions.

### Effects of desipramine on the genes involved in neurotransmitter transport

Among the genes involved in neurotransmission, microarray analysis revealed the upregulation of *Slc6a, Slc17a6, Slc6a11* (also *Glyt1, Vglut2 Gat3* respectively) and *Rab3. Glyt1* terminates the action of glycine through high affinity sodium-dependent reuptake into presynaptic terminals and plays a role in the regulation of glycine levels in NMDA receptor-mediated neurotransmission. *Vglut2* mediates the uptake of glutamate into synaptic vesicles at presynaptic nerve terminals of excitatory neural cells. *Gat3* terminates the action of GABA through high affinity sodium-dependent reuptake into presynaptic terminals and *Rab3* regulates synaptic membrane exocytosis. Because antidepressants inhibit GABA uptake, the upregulation of *Gat3* is an important factor in AD therapy. Tordera [64] showed that repeated treatment with fluoxetine, paroxetine or desipramine increased the abundance of *Vglut1* mRNA in the hippocampus, but did not increase the expression of *Vglut2* and *Vglut3* mRNA. The data obtained in the present study suggest that a course of AD drug treatment also increases the expression of *Vglut2*, another gene involved in the regulation of glutamate secretion, but only in vulnerable individuals, such as the HA mouse line.

### The impact of desipramine treatment on HA and LA mouse lines

The objective of the present study was to characterize the transcriptional response to chronic desipramine treatment in the hippocampus of mouse lines displaying robust differences in the behavioral responses to ADs. In a previous study, we showed that desipramine, venlafaxine and aminosenktide shortened the immobility period of HA mice in the forced swim and tail suspension tests, but were ineffective in the LA line [5-7]. Moreover, at the molecular level, the lines were characterized by differences in the basal transcriptomic profile in the hippocampus, which indicates that selective breeding for swim SIA affected many aspects of hippocampal neuron physiology, including metabolism, structural changes, and cellular signaling [8]. The genes involved in calcium signaling pathways are upregulated in LA mice, while HA mice are characterized by the robust upregulation of *Klf5* and genes encoding receptors for neurotensin and GABA [8]. *Klf5* encodes the KLF5 zinc finger protein, which act as transcription factors that bind to GC box promoter elements and activate the transcription of these genes, depending on the DNA methylation status in the promoter regions. In turn, we observed that chronic mild stress (CMS) generated a self-contained response within each mouse line [10]. In the HA line, CMS affected several genes involved in chromatin modifications, suggesting a role for the epigenetic regulation of the stress response in the HA line. KLF5 is primarily localized in the hippocampus, which is the principal source of glutamatergic neurotransmission. These findings suggest that KLF5 gene expression is involved in glutamatergic neurotransmission [65]. Altogether, the previous and current results on the hippocampal transcription patterns of the genes involved in calcium signaling, GABA neurotransmission, ion transport, and glutamate transporters suggest a functional endpoint for structurally unrelated antidepressants and explain the differences observed in the lines in response to antidepressants.

## Conclusions

The gene expression patterns during ADs treatment could be very complex. Nevertheless, our study revealed a number of novel potential targets for antidepressant therapy, such as the *Npy*, *Crhbp*, *Ttr*, *Igfbp2*, *Prlr*, and genes involved in neurogenesis that were not previously linked to antidepressant action. Potential drug targets against the detrimental effects of stress and depression include also glutamate transporters, GABA neurotransmission, and neuro- and axonogenesis, and suggest that pharmacotherapy might be genotype dependent. Single nucleotide polymorphisms (SNPs), epigenetic statuses or epimutations in the regulatory elements of the identified genes should be considered in the pharmacogenomics of antidepressants. Genome wide gene expression profiling using two lines of mice bred for stress reactivity facilitates the elucidation of broader mechanisms underlying antidepressants effects.

## Competing interests

The authors declare no competing interests.

## Authors’ contributions

PL carried out the RNA extractions, RNA quality assessment, microarray analysis, gene expression data extraction, and normalization, bioinformatics, drafted the manuscript and was responsible for the study concept and design. GRJ carried out the DMI treatment. JG performed the statistical analysis. AMS carried out the qPCR assays. MW dissected the brain structures. LZ participated in the design of the study. AHS supervised the project and contributed to the final draft of the paper. All authors read and approved the final manuscript.

## Supplementary Material

Additional file 1: Table S1 Primers. List of primers for investigated genes. Click here for file

Additional file 2: Table S2 HA_SAL_HP_vs_HA_DES_HP. Lists of probe set for genes with altered expression after chronic desipramine treatment in the hippocampus of HA mice. Click here for file

Additional file 3: Table S3 LA_SAL_HP_vs_LA_DES_HP. Lists of probe set for genes with altered expression after chronic desipramine treatment in the hippocampus of LA mice. Click here for file

Additional file 4: Table S4 HA_GO_FAT. Gene Ontology categories of differentially expressed genes in the hippocampus of saline-treated HA mice vs. desipramine-treated HA mice. Significantly enriched (p < 0.05) gene ontology (GO) categories of differentially expressed genes (DEGs) in the hippocampus of saline-treated HA mice vs. desipramine-treated HA mice. GO categories shown in the figure consist of biological processes, molecular functions, cellular components and biochemical pathways terms. Click here for file

Additional file 5: Table S5 LA_GO_FAT. Gene Ontology categories of differentially expressed genes in the hippocampus of saline-treated LA mice vs. desipramine-treated LA mice. Significantly enriched (p < 0.05) gene ontology (GO) categories of differentially expressed genes (DEGs) in the hippocampus of saline-treated LA mice vs. desipramine-treated LA mice. GO categories shown in the figure consist of biological processes, molecular functions, cellular components and biochemical pathways terms. Click here for file
